# Toxicity of Graphene Shells, Graphene Oxide, and Graphene Oxide Paper Evaluated with *Escherichia coli* Biotests

**DOI:** 10.1155/2015/869361

**Published:** 2015-06-16

**Authors:** Ludmila V. Efremova, Alexey S. Vasilchenko, Eduard G. Rakov, Dmitry G. Deryabin

**Affiliations:** ^1^Department of Microbiology, Orenburg State University, Pobedy Avenue 13, Orenburg 460018, Russia; ^2^Institute of Cellular and Intracellular Symbiosis, RAS, Pionerskaya Street 11, Orenburg 460000, Russia; ^3^D. Mendeleyev University of Chemical Technology, Miusskaya Square 9, Moscow 125047, Russia

## Abstract

The plate-like graphene shells (GS) produced by an original methane pyrolysis method and their derivatives graphene oxide (GO) and graphene oxide paper (GO-P) were evaluated with luminescent *Escherichia coli* biotests and additional bacterial-based assays which together revealed the graphene-family nanomaterials' toxicity and bioactivity mechanisms. Bioluminescence inhibition assay, fluorescent two-component staining to evaluate cell membrane permeability, and atomic force microscopy data showed GO expressed bioactivity in aqueous suspension, whereas GS suspensions and the GO-P surface were assessed as nontoxic materials. The mechanism of toxicity of GO was shown not to be associated with oxidative stress in the targeted *soxS*::*lux* and *katG*::*lux* reporter cells; also, GO did not lead to significant mechanical disruption of treated bacteria with the release of intracellular DNA contents into the environment. The well-coordinated time- and dose-dependent surface charge neutralization and transport and energetic disorders in the *Escherichia coli* cells suggest direct membrane interaction, internalization, and perturbation (i.e., “membrane stress”) as a clue to graphene oxide's mechanism of toxicity.

## 1. Introduction

Graphene is a two-dimensional one-atom thick layer of carbon packed into a honeycomb-like structure [1, 2]. Extremely thin carbon foils were predicted over more than 50 years ago [3] and the term of “graphene” was first introduced in 1987 [4] but true graphene was only created by Novoselov et al. in 2004 [5].

Since then, the technology of graphene and its derivatives have been developing actively [6]. The unique physical properties of graphene, such as its exceptional mechanical strength, thermal stability, and high electrical conductivity, attract attention in various fields of science and technology [7–9]. The growing interest in graphene-family nanomaterials (GFNs) is driving the study of their biological activity as well. It is necessary to evaluate environmental risks of graphene-containing technological objects to biological systems [10], as it is for other carbon-based nanomaterials [11], in particular, fullerenes [12] and nanotubes [13]. Increasing information about graphene toxicity shows that its number of layers, lateral size, stiffness, hydrophobicity, surface functionalization, and dose are important [1, 14–17]. However, the toxicity and biocompatibility of GFNs are still debated [18].

Evaluating graphene's activity against bacteria is an important step to understanding GFNs' bioactivity. These model organisms are responsive and sensitive to various damaging factors, and their physiological manifestations allow understanding of toxicity mechanisms. In 2010, Akhavan and Ghaderi [19] first described the toxic effect of GFNs against several bacterial species and also showed that graphene oxide was more active compared to a pristine graphene sample. Since then, the toxicity of GFNs against bacteria has been studied extensively (e.g., an ISI Web of Knowledge topic search on 25/06/2014 gave 168 hits for “graphene” and “bacteria”), but the results in dozens of publications are contradictory. In particular, this applies to so-called “graphene paper” [20], which showed an absence of effects in some studies [21] while in other cases strong antibacterial activity was reported [22, 23].

*Escherichia coli* biotests are very attractive for solving this problem. These bacteria are a wide-spread model organism in modern toxicology because well characterized physiology and ease of genetic manipulation [24]. In particular, the bioassays based on recombinant luminescent light-off and light-on* E. coli* strains gave the possibility of obtaining the detailed information about the biological activity of the tested compounds in real time manner [25]. Previously, in our studies the bioluminescence inhibition test has been used to assess the toxicity of wide range of carbon-based nanomaterials [26]. In turn, in a recent study by Jia et al. [27] the inducible luminescent* E. coli* strains have been used to assess the toxicity mechanisms of various carbon nanomaterials including graphene nanosheets.

In the continuation of this research, the aim of this study was to evaluate some graphene-family nanomaterials using the luminescent* Escherichia coli* biotests and additional bacterial-based assays which together reveal the GFNs toxicity and bioactivity mechanisms.

## 2. Experimental

### 2.1. Graphene-Family Nanomaterials

The graphene-family nanomaterials used in this work included graphene shells (GS), graphene oxide (GO), and graphene oxide paper (GO-P).

**Figure 1:**
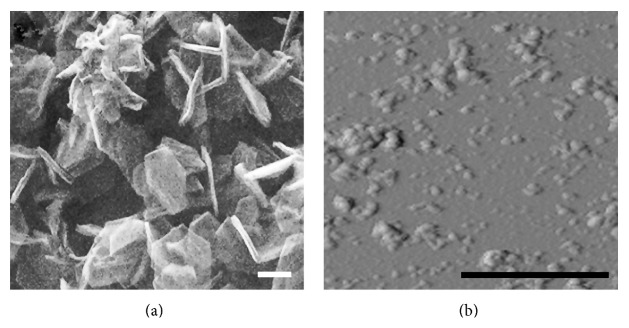
High resolution scanning electron microscopy image of the graphene shells (a) and atomic force microscopy image of graphene oxide particles (b). Scale bar: 500 nm.

GS were synthesized through methane pyrolysis at 800°C on MgO plates with hexagonal habitus and approximate size of hexagon edge 700 nm and average thickness of 115 nm [28]. After MgO dissolution in diluted hydrochloric acid the hollow hexagonal aggregates 95 nm in thickness consisting of separate particles which contain several graphene layers were obtained (Figure 1(a)). The specific surface area of the synthesized material was 1585 m^2^/g, corresponding to a bilayer of graphene particles. GO were prepared by GS anodic oxidation in sulfuric acid and subsequent oxidation with a mixture of sulfuric and nitric acids under heating and microwave irradiation [29]. Atomic force microscopy of the GO particles (Figure 1(b)) showed significant fragmentation of the plate-like shells as an indirect result of the oxidation process.

**Figure 2:**
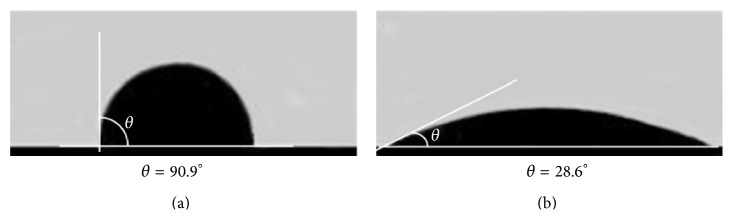
Examples of typical contact angles of the graphene-family nanomaterials surface: (a) graphene shells and (b) graphene oxide.

GFNs covering solid surfaces were used for quantitative measurement of wetting by a polar liquid and work of adhesion (*W*_*a*_) calculations. Briefly, a 2 *μ*L drop of deionized water was placed on the surface of a preformed GFN at 20 ± 1°C and images were obtained instantaneously using a digital camera (Figure 2). From the images the values of the static contact angles were determined and *W*_*a*_ values were calculated by the Young-Dupre equation as *W*_*a*_ = *σ* × (1 + cos*θ*), where *σ* is the surface tension of water at 20°C, taken as 72.86 × 0.001 N/m, and *θ* is the average value of the static contact angle. This integral parameter characterizing GFNs' hydrophobicity/hydrophilicity was 74.4 ± 1.5 N/m and 137.1 ± 2.3 N/m for GS and GO, respectively.

**Figure 3:**
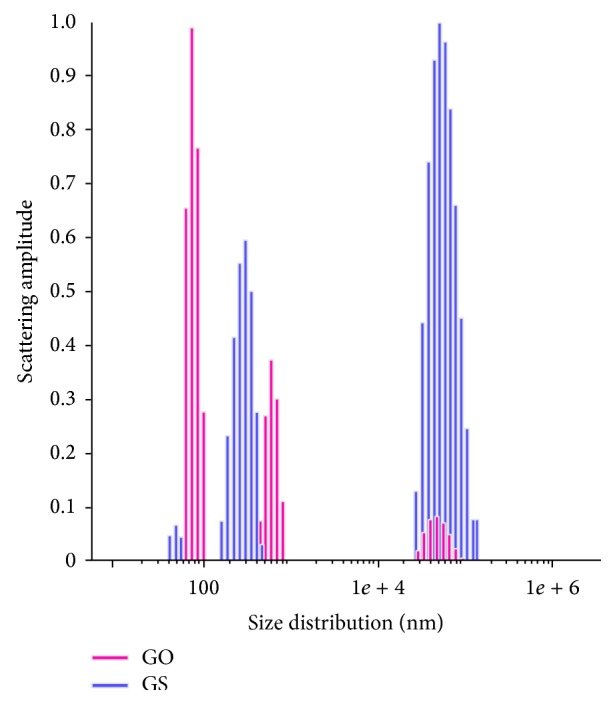
Dynamic light scattering profiles for aqueous graphene shells (GS) and graphene oxide (GO) suspensions.

Aqueous suspensions of GS and GO (from 10^−3^ to 2 × 10^−5^ mg/L) were prepared in deionized water in vials, vigorously mixed and sonicated in an ultrasonic bath (Sapfir, Russia) at 35 kHz and a specific power of 30 W × dm^−3^ for 30 min. The suspensions were then incubated for about 2 hours at 20°C, thus allowing the colloidal systems to reach equilibrium. Dynamic light scattering measurements were performed using the Photocor Compact-Z (Russia). The measured autocorrelation functions were analyzed and the effective hydrodynamic radius (RH) was calculated according to the Einstein-Stokes Relation using the software supplied with the instrument (Figure 3).

The GS aqueous suspension was polydisperse and consisted of three fractions: about 70% were large aggregates with RH values of 67 ± 30 *μ*m, and only 10% were particles with RH = 45 ± 27 nm. In turn, 66% of the aggregates in the GO suspension were characterized by an RH of 80 ± 3.7 nm, whereas <10% of the aggregates were large particles. Thus, the preliminary characterization of the GFNs showed them to be nano- and microscale carbon compounds, where GS is composed of hydrophobic plate-like shells which are poorly dispersible in polar solvents, whereas GO consists of hydrophilic fragmentized particles which form a finely dispersed aqueous suspension.

A GO suspension was used for graphene oxide paper production. The GO-P was made by spraying the graphene oxide shell colloid on a white cellulose chlorine-free SvetoCopy paper 80 g/m^2^ followed by air drying. This method gave a uniformly coated, well-wetted, and nonwaterproof surface of GFN material.

### 2.2. Preparation of Bacterial Strains and Cells

The experiments involving bacterial cells for GFN bioactivity assessments were performed using three* Escherichia coli* K12-based* lux*-biosensors (Table 1).

The first was the commercially available* E. coli* K12 TG1 strain containing a recombinant plasmid with the* luxCDABE* operon of* Photobacterium leiognathi* cloned under the* lac* promoter, which shows strong constitutive light emission under standard cultivation conditions. Inhibition of its bioluminescence is likely due to the toxicity associated with reducing bacterial metabolite levels or cell death, because light production requires active cell metabolism. Lyophilized* E. coli* K12 TG1* lac::lux* cells purchased from “Immunotech” (Russia) were rehydrated with cold distilled water to a concentration of 3.7 × 10^8^ colony-forming units (CFU) per 1 mL, exposed for 30 min at 2–4°C, and then the temperature was raised up to 25°C before use.

Two* E. coli* K12 MG1655-based strains carried recombinant plasmids containing a transcriptional fusion of host* soxS* or* katG* promoters to a truncated* Photorhabdus luminescens,* the* luxCDABE* operon. These inducible bioluminescent strains exhibited low basal light emission that increases significantly during an oxidative stress response as a consequence of simultaneous transcription of the* soxS* or* katG*-gene and* lux*-genes fusions [30]. The bacteria were grown on LB-agar (Sigma-Aldrich, USA) supplemented with 100 *μ*g/mL of ampicillin for 18–24 hours at 37°C, after which the cells were transferred to fresh LB-broth and harvested at an optical density of 0.1 absorption units at 545 nm, corresponding to a concentration of 2.5 × 10^7^ colony-forming units (CFU) per 1 mL.

The nonluminescent* E. coli* K12 TG1 strain was used as a control for fluorescence and some additional bioassays (see the following).

### 2.3. Bioluminescent Assays

Light-off (bioluminescence inhibition) and light-on (bioluminescence induction) assays were used for GFN bioactivity evaluations.

To assess the GFNs' toxicity we used a previously described version of bioluminescent analysis for carbon-based nanomaterials [26]. Briefly, sonicated aqueous GS and GO suspensions were added to the wells of a “Microlite 2+” microplate with nontransparent side walls (Thermo, USA), wherein they were further doubly diluted in sterile distilled water, from 1 : 1 to 1 : 1024, up to a final volume of 50 *μ*L. To the filled wells was then added 50 *μ*L of a previously prepared suspension of constitutively luminescent* E. coli* K12 TG1* lac::lux* cells. The key feature of the present experiment was a set of serial dilutions of the GFNs, so we could investigate various ratios of nanoparticles to cells. Wells filled with sterile distilled water and containing an appropriate amount of bacterial biosensor were used as controls.

To assess the graphene oxide paper we used an original variation that provided placement of an intact sterile disc (Himedia, India) into the wells, followed by the placement of GO-P discs as a second layer and then applying 50 *μ*L of the constitutively luminescent bacterial suspension to the surface. Second layers of white and black paper were used as the controls.

Bioluminescence measurements were carried out using an LM-01T microplate luminometer (Immunotech, Czech Republic), which dynamically registered the luminescence intensity of the samples for 180 min, estimated in relative light units (RLU). The data were analyzed using KILIA graphing software. To quantify the bioluminescence inhibition index (*I*) due to GFN toxicity we used the algorithm *I* = RLU*c*_0_ × RLU*t*_*n*_/RLU*c*_*n*_ × RLU*t*_0_, where *c* and *t* are the RLU values of the control and test samples at the 0th and *n*th minute of measurement. Based on these indexes, we calculated the EC50 toxicological parameters, that is, the GFN concentrations that cause 50% inhibition of constitutive bioluminescence.

The bioluminescence induction assay was conducted as follows:* E. coli* K12 MG1655* soxS::lux* or* E. coli* K12 MG1655* katG::lux* were washed from the cultivation media and then adjusted with 0.85% NaCl in water to obtain a final optical density at 545 nm of 0.1; a 50 *μ*L aliquot of the culture was then added to each well filled with a GO suspension as described above. Solvent only and untreated cells were used as the negative control samples. Paraquat (final concentrations 12.5–0.00305 mmol) and H_2_O_2_ (0.00938–0.00002%) were added to the positive control wells as standards for oxidative stress induction in* soxS::lux* and* katG::lux*, respectively. After 15, 30, or 60 min, 100 *μ*L of fresh LB-broth was added to each well and the plates were incubated at 30°C in a LM-01T luminometer. Bioluminescence was monitored every 3 min for at least 120 min without shaking. The results were presented as induction coefficients, defined as the relative light units (RLU) of an induced sample divided by that of the untreated control samples.

### 2.4. Fluorescent Bacterial Staining

A two-component fluorescent Live/Dead Baclight kit L-13152 (Molecular Probes, USA) was used to evaluate membrane integrity in nonluminescent* E. coli* K12 TG1 cells treated with GFNs. This direct-count assay utilizes two nucleic acid staining dyes, green-fluorescent SYTO 9 (excitation/emission maxima 480/500 nm), which labels all bacteria, and red fluorescent propidium iodide, PI (490/635 nm), which penetrates damaged membranes only, causing a reduction in SYTO 9 fluorescence when both dyes are present. Thus, bacteria with intact membranes stain fluorescent green, whereas cells with increased passively permeable membranes stain fluorescent red.

Suspensions containing log-phase* E. coli* K12 TG1 cells (OD_540_ = 0.01) in distilled water were mixed with GS and GO suspensions placed on the GO-P surface and incubated for 60, 120, and 180 minutes in a moist chamber. For dyeing, a portion of a liquid sample or paper fragments were transferred to nonfluorescent microscopy glass and subjected to one-step staining according to the manufacturer's recommendation. The bacteria's colors were observed in a Mikromed-3-Lum fluorescent microscope (Russia) equipped with filter sets useful for simultaneous viewing of the SYTO 9 and PI stains; images for cell quantification were then acquired with a digital camera.

### 2.5. Atomic Force Microscopy

Visualization of contacts between GFNs and bacterial cells was performed using an atomic force microscope SMM-2000 (Proton-MIET, Russia) as described previously [31]. Briefly, aliquots (20 *μ*L) of intact bacterial cells, alone or mixed with GS or GO suspensions, were applied to freshly prepared mica, and intact bacterial cells were applied to the GO-P surface. The samples were incubated at 93% relative humidity and 20–22°C and scanned in a contact mode using V-shaped silicon nitride cantilevers (MSCT-AUNM, Veeco Instruments Inc., USA) with a spring constant of 0.01 N/m and a tip curvature of 10 nm. Quantitative morphometric analysis of the images was performed using the standard software provided with the instrument.

### 2.6. Efflux of Intracellular DNA Content

The plasmid-containing* E. coli* K12 TG1* lac::lux* strain was cultured in mid-exponential growth phase with ampicillin and then separated from its cultivation medium and incubated with aqueous GO suspensions at concentrations of 1.25 × 10^−4^, 5 × 10^−4^, and 2 × 10^−3^ mg/L for 60 min, or with solvent only (negative control) or 0.5% SDS solution, which gives rapid 100% lysis of all bacterial cells (positive control). After centrifugation at 14000 g for 15 min (Eppendorf, Germany), the concentration of total DNA in the supernatant was analyzed by fluorescence spectroscopy (Solar CM2203, Belarus) using ethidium bromide as a fluorescent dye (excitation/emission maxima 285/605 nm). Salmon sperm DNA was used as an external standard (0–3 *μ*g/mL).

### 2.7. Zeta Potential Measurement

The zeta potential (membrane surface charge) of* E. coli* K12 TG1 cells was determined using Photocor Compact-Z (Russia) equipment. A bacterial suspension (3.7 × 10^8^ CFU/mL) was mixed with serially diluted GO aqueous suspensions to a final concentration from 3.1 × 10^−5^ to 5 × 10^−4^ mg/L. Measurements were carried out in triplicate for two samples at 60, 120, and 180 minutes. Zeta potentials were calculated from the electrophoretic cell mobility data obtained from Smoluchowski's equation.

### 2.8. Statistics

The results were processed statistically by the method of variance using the software package Statistica V8 (StatSoft Inc., United States).

## 3. Results and Discussion

### 3.1. Comparative Evaluation of GFNs against* Escherichia coli* Cells

#### 3.1.1. GFN Toxicity Assessment Using a Bacterial Bioluminescence Inhibition Assay

**Figure 4:**
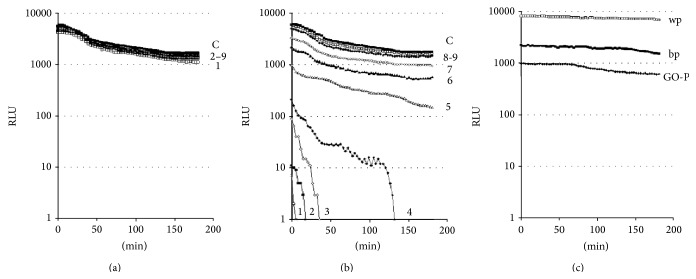
The time course of* E. coli* K12 TG1* lac::lux* luminescence during contact with graphene shells (a) and graphene oxide (b) aqueous suspensions, or graphene oxide paper and control paper (c). Ordinate-luminescence level, RLU; abscissa-time measurement, min. Designations: 1: 10^−3^ mg/L; 2: 5 × 10^−4^ mg/L; 3: 2.5 × 10^−4^ mg/L; 4: 1.25 × 10^−4^ mg/L; 5: 6.25 × 10^−5^ mg/L; 6: 3.13 × 10^−5^ mg/L; 7: 1.56 × 10^−5^ mg/L; 8: 7.81 × 10^−6^ mg/L; 9: 3.91 × 10^−6^ mg/L; C: control; wp: white paper, bp: black paper, GO-P: graphene oxide paper.

Measurement of* E. coli* K12 TG1* lac::lux* luminescence in contact with GFNs revealed rapid light inhibition in the first second of instrument registration (Figure 4). The GS suspension showed a less pronounced effect, in contrast to the GO suspension, which dose-dependently decreased the luminescence intensity. In our experience [26], this “false” effect was determined by optical distortion in GFNs suspensions not being associated with carbon-based nanomaterials bioactivity.

A similar result was apparent during the contact between* E. coli* K12 TG1* lac::lux* and the paper samples. The luminescence intensity decreased significantly in the first second when the biosensor was applied on GO-P or to a control black paper, while the luminescence intensity with the control white paper was high due to this material's minimal light absorption and maximal light reflection.

Continued luminescence measurement showed different light intensity changes due to biosensor contact with the GS suspension and GO-P on the one hand and the GO suspension on the other. The poorly dispersible GS suspension was evaluated as nontoxic because of the absence of significant luminescence inhibition (Figure 4(a)). In contrast, the finely dispersed GO suspension led to inhibition of luminescence and in some cases to zero luminescence, depending on the concentration and with increasing time (Figure 4(b)). This effect was interpreted as “true” toxicity of graphene oxide against the* E. coli* K12 TG1* lac::lux* biosensor. In the current system containing 3.7 × 10^8^ CFU/mL cells, GO toxicity was characterized by EC50 values of 1.13 ± 0.05 × 10^−4^, 1.04 ± 0.03 × 10^−4^, and 7.92 ± 0.24 × 10^−5^ mg/L after 60, 120, and 180 min of measurement, respectively. In turn, the initial decrease in* E. coli* K12 TG1* lac::lux* luminescence on the GO-P surface, accompanied by a very slow further decline in light production (Figure 4(c)), led to the evaluation of this material as nontoxic.

Thus, the bioluminescence inhibition assays showed a GFN's bioactivity was dependent on its hydrophilicity/dispersivity (in the case of the GS versus GO comparison) and on the type of interaction with the bacterial biosensor (the GO suspension was toxic while the surface of the GO-P was not).

The second step in assessing graphene oxide toxicity against the* E. coli* K12 TG1* lac::lux* strain examined various GO : cell suspension ratios. This approach allowed us to analyze the relationships between the surface areas, which were calculated based on bacterial CFU per 1 mL and DLS-data, respectively.

**Figure 5:**
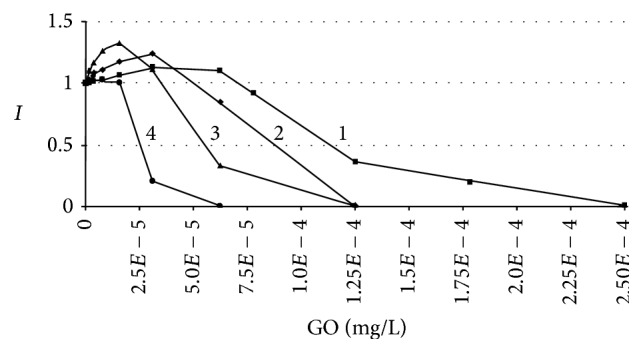
The dose-response curves described the changes in the bioluminescence inhibition indexes (*I*) caused by differing graphene oxide (GO) content and varying bacterial cell number in the sample: 1–3.70 × 10^8^ CFU per 1 mL; 2–1.85 × 10^8^ CFU per 1 mL; 3–9.25 × 10^7^ CFU per 1 mL; 4–4.62 × 10^7^ CFU per 1 mL.

The bioluminescence inhibition assay showed that GO toxicity was dependent upon the content of bacterial cells in the sample (Figure 5) whereby the EC50 values decreased with decreasing the CFU number (Table 2). At the 60th min of measurement these values varied from 1.13 ± 0.05 × 10^−4^ to 2.55 ± 0.10 × 10^−5^ mg/L following twofold bacterial cell dilutions. These data showed the need to cover the bacterial surface with a certain number of GO particles in order for the toxic effect to be apparent. So the calculation of GO : cell ratios gave a stable value of 0.5 ± 0.05 *μ*m^2^ GO surface for 1 *μ*m^2^ bacterial surface as necessary for 50% bioluminescence inhibition. Prolonging the bioluminescence measurement to 120 and 180 min revealed an ongoing toxic effect that is evident in Table 2 by the decreasing EC50 values. This observation shows the dose-dependency as well as time-dependency of GO toxicity in aqueous suspension.

#### 3.1.2. GFN Bioactivity in a Fluorescent Cell Assay

**Figure 6:**
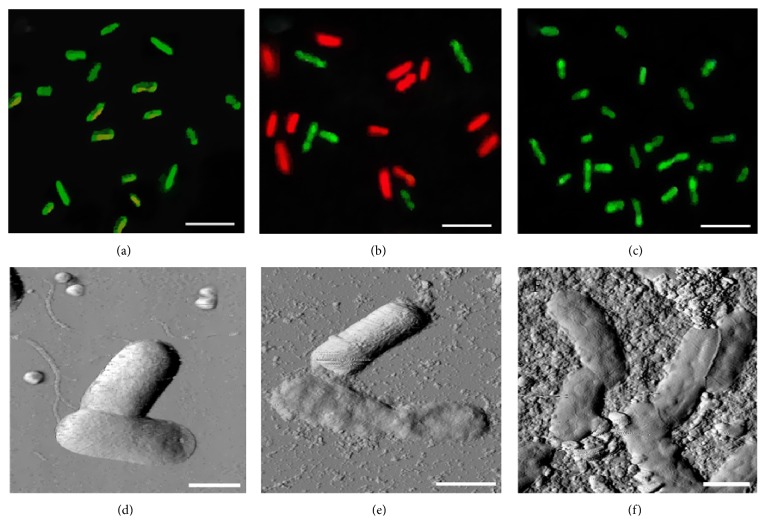
Fluorescent microscopy images ((a)–(c)) and atomic force microscopy images ((d)–(f)) of* E. coli* K12 TG1 cells treated with graphene shells suspension at 10^−3^ mg/L for 180 min ((a), (d)) and graphene oxide suspension at 1.25 × 10^−4^ mg/L for 180 min ((b), (e)) and placed on the surface of graphene oxide paper for 60 min ((c), (f)). Scale bar: 5 *μ*m ((a)–(c)); 1 *μ*m ((d)–(f)).

Two-component differential fluorescent labeling of the nonluminescent* E. coli* K12 TG1 strain detected 99.9 ± 0.1% green-fluorescent cells in both samples treated with GS and GO-P (Figures 6(a) and 6(c)) indicating intact membranes, consistent with the bioluminescent assay results. In contrast, bacterial cells treated with GO suspensions became red-fluorescent after 60 min in a dose-dependent manner, from 48.8 ± 1.9% (at 1.25 × 10^−4^ mg/L GO) to 93.0 ± 2.8% (at 5 × 10^−4^ mg/L GO) because of membrane damage that allowed the fluorescent PI dye to penetrate (Figure 6(b)). Prolonging the exposure to 180 min increased the red-fluorescent cell fraction to 73.9 ± 2.2%–94.7 ± 3.3%. These data suggested GO bioactivity against the* E. coli* cells and showed increased passive membrane permeability and bioluminescence inhibition as simultaneous toxicity indicators. The data from the bioluminescent and fluorescent assays were nonidentical but well correlated, as will be discussed below (see Section 3.2.3).

#### 3.1.3. AFM-Evaluation of GFN and* Escherichia coli* Cell Interactions

*E. coli* K12 TG1 cells treated with GS and GO suspensions or applied on the surface of GO-P were transferred to mica and visualized by AFM-microscopy (Figures 6(d)–6(f)).

This study did not reveal any differences in GS-treated cells in shape and size (1.22 *μ*m^3^ in volume) compared to control intact bacteria. Cell contact with GS was also not detected (Figure 6(d)), despite large 0.5–3.0 *μ*m aggregates on the mica surface around the cells being visualized. In contrast,* E. coli* K12 TG1 cells incubated with GO suspensions had significantly altered morphology (Figure 6(e)). The bacterial cell surface was covered with numerous 67 ± 13 nm particles that were apparent around the cells too and corresponded well to the GO particle sizes revealed by dynamic light scattering (see Section 2.1). As a result, the roughness of the GO-covered bacterial surface was more than twofold greater in comparison with the control cells (*P* < 0.001). The volume (0.81 *μ*m^3^, *P* < 0.01) of GO-treated cells also differed from those of control cells. Moreover, some GO-treated cells changed their morphology more significantly and were visualized spread-eagled on the mica surface, characteristic of membrane permeability damage. At the same time,* E. coli* K12 TG1 cells lying on the surface of the GO-P were indistinguishable from the control samples in terms of volume (1.12 *μ*m^3^), as well as not showing any AFM-detected lesions (Figure 6(f)).

Thus three assays, including bioluminescence, fluorescence, and AFM, showed the toxicity of a GO suspension against* Escherichia coli* cells and established the absence of toxicity of a GS suspension or GO-covered paper. The obtained results were the basis for further research into the mechanisms of graphene oxide toxicity.

### 3.2. Evaluation of GO Toxicity Mechanisms against* Escherichia coli* Cells

#### 3.2.1. GO Validation as a Potential Oxidative Stress Inducer in Lux-Biotests

The* E. coli* K12 MG1655* soxS::lux* and* E. coli* K12 MG1655* katG::lux* strains were used in a bioluminescence induction assay for oxidative stress as reflected in increased light emission.

**Figure 7:**
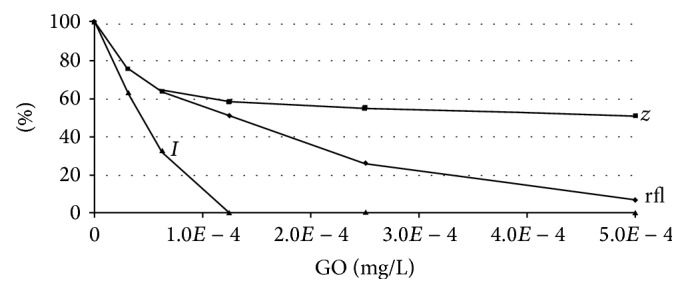
The relationship between* E*.* coli* K12 TG1 cells' zeta potential values (*z*), red fluorescent labeling (rfl), and bioluminescence inhibition indices (*I*) depended on the graphene oxide (GO) content in the suspensions.

The luminescent response of these strains can be verified with model oxidants such as paraquat - 1,1′-dimethyl-4,4′-bipyridinium dichloride (Sigma, USA), causing electron transfer from bacterial respiratory chains onto molecular oxygen with intracellular superoxide-anion production, or in the presence of exogenous hydrogen peroxide (Figure 7).* E. coli* K12 MG1655* soxS::lux* cells treated with paraquat concentrations as low as 0.00305 mmol exhibited much stronger dose-dependent luminescence as a consequence of simultaneous transcription of the* soxS*-gene and* soxS::lux* genes fusions. A peak 8.7-fold increase in bioluminescence was detected with 0.04883 mmol paraquat treatment, whereas the highest concentrations of the inducer resulted in inhibition of luminescence because of their extreme toxic effect. In turn,* E. coli* K12 MG1655* katG::lux* was activated by hydrogen peroxide at a concentration of 0.00002% and achieved a maximal 17.6-fold induction at 0.00469%.

Bacterial luminescence measurement in the presence of the GO suspension did not show significant induction coefficients. The 1.2–1.5-fold light intensity growth only has been recorded at subtoxic GO concentrations (1.95 × 10^−6^–10^−3^ mg/L). In the current experimental context it was unlikely the stress response, more likely it was a recovery of bioluminescent reaction after nutrient media supplementation than stress response. In turn, GO concentrations of 2 × 10^−3^ mg/L or more were toxic and resulted in irreversible bacterial luminescence inhibition in the* E. coli* K12 MG1655* soxS::lux* and* E. coli* K12 MG1655* katG::lux* strains, just as was shown in the* E. coli* K12 TG1* lac::lux* luminescence assay (see Section 3.1.1). Thus the mechanism of GO toxicity is not associated with oxidative stress in the targeted* E. coli* cells.

#### 3.2.2. Intracellular DNA Efflux Measurement in GO-Treated* Escherichia coli* Cells

The toxicity caused by cellular membrane damage was further verified by measuring intracellular DNA efflux by plasmid-containing* E. coli* K12 TG1 cells exposed to GO. The DNA concentration in intact control supernatants was undetectable using a fluorescence spectroscopy assay with ethidium bromide as a fluorescent dye. In contrast, DNA was easy to detect in the supernatant of* E. coli* treated with SDS and was quantified as 3 *μ*g/mL according to DNA standards. In turn, DNA levels in supernatants of cells exposed to GO at concentrations of 1.25 × 10^−4^–2 × 10^−3^ mg/L for 60–180 min were similar to those of the intact control, whereas both the luminescent and fluorescent assays showed a toxic effect. Thus we report that GO-induced membrane damage did not lead to cell lysis, reflected in an efflux of biopolymers (including plasmid DNA), but their increased passive permeability was rather limited to small molecules (including fluorescent dyes).

#### 3.2.3. Effect of GO Treatment on Zeta Potential Properties of* Escherichia coli* Cells

Zeta potential studies were carried out to reveal the effect of GO on the membrane surface charge in* E*.* coli* K12 TG1 cells. The control bacterial sample displayed a zeta potential value of −31.31 ± 0.94 mV, which gradually decreased to −24.19 ± 0.72 mV during dynamic measurement. Upon the addition of increasing GO concentrations, the* E*.* coli* zeta potential values decreased slightly upon initial contact and then the membrane surface charge was progressively neutralized in a dose-dependent manner from 60 min (Figure 7) until 180 min. Finally, the GO concentration required for complete zeta potential neutralization was 2.50 × 10^−4^ mg/L, whereas concentrations in the range of 3.13 × 10^−5^–1.25 × 10^−4^ mg/L were sufficient to promote zeta potential values from −18.49 ± 1.49 mV to −9.44 ± 0.02 mV. These data show that zeta potential neutralization is an important result of GO nanoparticles' interaction with bacterial surfaces, having the negative charge originating from outer lipopolysaccharide molecules and using this electrical potential in a membrane-associated energetic process.

Interestingly, measurements of* E*.* coli* cells treated with increasing GO concentrations established a correlation between zeta potential neutralization and bioluminescence inhibition values at 60 min (*r* = 0.973, *P* < 0.01). This can be interpreted as GO targeting of electrical potential across cell membranes that affected the energy metabolism available for biochemical reactions in a bacterial cell, including those required for energetic bioluminescent substrates, wherein the bacterial bioluminescence inhibition assay was the most sensitive, which allows us to recommend it to assess a GFN's toxicity. The two-component fluorescent data evaluating cell membrane permeability and zeta potential measurement also correspond well (*r* = 0.943, *P* < 0.01), indicating a dual violation of bacterial membrane functions. The similar trends for these parameters (Figure 7) suggest membrane perturbation (i.e., “membrane stress”) is a clue to the mechanism of grapheme oxide toxicity, in which GO adhesion and internalization processes trigger transport and energetic disorders.

## 4. Conclusion

The toxicity of graphene-family nanomaterials, including pristine grapheme, against bacteria was first described in 2010 [19], but subsequent results have been contradictory and understanding GFNs' mechanisms of toxicity is incomplete [18, 32]. In this work we studied graphene shells synthesized using an original methane pyrolysis method [28], a graphene oxide derivative [29], and graphene oxide paper in several* Escherichia coli* biotests sufficient to reveal the GFNs' toxicity and bioactivity mechanisms.

Combining a bioluminescence inhibition assay and two-component fluorescence in order to evaluate cell membrane permeability with atomic force microscopy, there was an absence of detectable bioactivity for GS. Although the GS sample used differed from canonical graphene and contained several (usually two) layers, the data clearly show this type of GFN is nontoxic. We attribute this result to GS not being well dispersed in water or other polar solvents [33], whereas this condition is a prerequisite for bioassays as in real biological systems.

The oxidation reaction led to graphene fragmentation as well as increased hydrophilicity, and the highly dispersible GO particles interacted strongly with the bacterial cell surface and caused a time- and dose-dependent toxic effect. Thus we confirm the high toxicity of graphene oxide against bacterial cells compared to its nonreduced counterpart [14, 19], determined by significant changes in its physicochemical properties. The revealed GO toxicity mechanism can be summarized as follows: (i) substantial covering of the bacterial surface by GO is a necessary prerequisite for the manifestation of bioactivity; (ii) GO adhesion causes bacterial surface charge neutralization; (iii) GO internalization by the cell membrane leads to increased passive membrane permeability, including PI dye; (iv) energetic processes in GO-treated cells were violated, resulting in bioluminescence inhibition as the most sensitive parameter for carbon-based nanomaterial toxicity evaluation. In contrast, we did not find significant mechanical disruption of GO-treated cells with the release of intracellular DNA into the environment or oxidative stress in the targeted bacterial cells [34, 35], indicating “membrane stress” as a clue to the mechanism of graphene oxide toxicity.

The GO-covered paper surface was nontoxic for* Escherichia coli* cells, in agreement with [21, 36] but in contradiction to [22, 23], which showed high antibacterial activity by graphene paper. In our opinion the biological inertness of GO-P was determined by its targeting only one-half of the bacterial surface, whereas the intact membrane provided a sufficient surface for vital functions. Moreover, in contrast to pristine graphene, graphene oxide is not electrically conductive, while charge transfer is thought to be a leading cause of the antibacterial property of graphene film [37]. Finally, the absence of impurities or metal nanoparticles [38, 39] also resulted in the nontoxicity of the used GO-P sample.

In summary, our results showed the importance of the physicochemical properties of graphene-family nanomaterials, as well as the quality of their suspensions or spatial surface organization for their toxicity against* Escherichia coli* cells. We revealed the expressed graphene oxide bioactivity in aqueous suspensions, whereas graphene shells and graphene oxide paper were determined to be nontoxic materials. The obtained result requires the regulation of GO's presence in the environment [24, 40], as well as confirming that the antibacterial property of GO has the potential to be useful [19, 38]. In pursuing biomedical applications, great care must be taken to ensure the toxicity or safety of each GFN against bacterial and eukaryotic cells is well characterized and understood.

## Figures and Tables

**Table 1:** *Escherichia coli *strains used in this study.

Host strain	Plasmid description	Type of light emission	Reference
*E. coli* K12 TG1	*lac* ′::*luxCDABE *	Constitutive	[26]
*E. coli* K12 MG1655	*soxS* ′::*luxCDABE *	Inducible, responsive to oxidative stress	[30]
*E. coli* K12 MG1655	*katG* ′::*luxCDABE *	Inducible, responsive to oxidative stress	[30]
*E. coli* K12 TG1	—	Nonluminescent	Collection strain

**Table 2:** Dose-dependent and time-dependent GO aqueous suspensions toxicity in bacterial bioluminescence inhibition assay characterized by EC50 value (mg/L content) at varying bacterial cell number in the sample.

*E. coli* K12 TG1 *lac*::*lux*, CFU per 1 mL	Time measurement		
	60 min	120 min	180 min
3.70 × 10^8^	1.13 ± 0.05 × 10^−4^	1.04 ± 0.03 × 10^−4^	7.92 ± 0.24 × 10^−5^
1.85 × 10^8^	8.81 ± 0.26 × 10^−5^	8.20 ± 0.33 × 10^−5^	5.67 ± 0.17 × 10^−5^
9.25 × 10^7^	5.57 ± 0.22 × 10^−5^	5.04 ± 0.23 × 10^−5^	3.88 ± 0.12 × 10^−5^
4.62 × 10^7^	2.55 ± 0.10 × 10^−5^	2.49 ± 0.10 × 10^−5^	2.28 ± 0.09 × 10^−5^

## References

[1] Zhang H., Grüner G., Zhao Y. (2013). Recent advancements of graphene in biomedicine. *Journal of Materials Chemistry B*.

[2] Geim A. K., Novoselov K. S. (2007). The rise of graphene. *Nature Materials*.

[3] Boehm H. P., Clauss A., Fischer G. O., Hofmann U. (1962). Das Adsorptionsverhalten sehr dunner Kohlenstoffolien. *Zeitschrift für Anorganische und Allgemeine Chemie*.

[4] Hamwi A., Mouras S., Djurado D., Cousseins J. C. (1987). New synthesis of first stage graphite intercalation compounds with fluorides. *Journal of Fluorine Chemistry*.

[5] Novoselov K. S., Geim A. K., Morozov S. V. (2004). Electric field in atomically thin carbon films. *Science*.

[6] Novoselov K. S. (2014). Technology: rapid progress in producing graphene. *Nature*.

[7] Rao C. N. R., Sood A. K., Subrahmanyam K. S., Govindaraj A. (2009). Graphene: the new two-dimensional nanomaterial. *Angewandte Chemie International Edition*.

[8] Guo S., Dong S. (2011). Graphene nanosheet: Synthesis, molecular engineering, thin film, hybrids, and energy and analytical applications. *Chemical Society Reviews*.

[9] Jang H., Lee J., Min D.-H. (2014). Graphene oxide for fluorescence-mediated enzymatic activity assays. *Journal of Materials Chemistry B*.

[10] Bradley D. (2012). Is graphene safe?. *Materials Today*.

[11] Tang A. C. L., Hwang G.-L., Tsai S.-J. (2012). Biosafety of non-surface modified carbon nanocapsules as a potential alternative to carbon nanotubes for drug delivery purposes. *PLoS ONE*.

[12] Mashino T., Nishikawa D., Takahashi K. (2003). Antibacterial and antiproliferative activity of cationic fullerene derivatives. *Bioorganic & Medicinal Chemistry Letters*.

[13] Jia G., Wang H., Yan L. (2005). Cytotoxicity of carbon nanomaterials: single-wall nanotube, multi-wall nanotube, and fullerene. *Environmental Science and Technology*.

[14] Liu S., Zeng T. H., Hofmann M. (2011). Antibacterial activity of graphite, graphite oxide, graphene oxide, and reduced graphene oxide: membrane and oxidative stress. *ACS Nano*.

[15] Santos C. M., Tria M. C. R., Vergara R. A. M. V., Ahmed F., Advincula R. C., Rodrigues D. F. (2011). Antimicrobial graphene polymer (PVK-GO) nanocomposite films. *Chemical Communications*.

[16] Schinwald A., Murphy F. A., Jones A., MacNee W., Donaldson K. (2012). Graphene-based nanoplatelets: a new risk to the respiratory system as a consequence of their unusual aerodynamic properties. *ACS Nano*.

[17] Sanchez V. C., Jachak A., Hurt R. H., Kane A. B. (2012). Biological interactions of graphene-family nanomaterials: an interdisciplinary review. *Chemical Research in Toxicology*.

[18] Bianco A. (2013). Graphene: safe or toxic? the two faces of the medal. *Angewandte Chemie International Edition*.

[19] Akhavan O., Ghaderi E. (2010). Toxicity of graphene and graphene oxide nanowalls against bacteria. *ACS Nano*.

[20] Dikin D. A., Stankovich S., Zimney E. J. (2007). Preparation and characterization of graphene oxide paper. *Nature*.

[21] Park S., Mohanty N., Suk J. W. (2010). Biocompatible, robust free-standing paper composed of a TWEEN/graphene composite. *Advanced Materials*.

[22] Hu W., Peng C., Luo W. (2010). Graphene-based antibacterial paper. *ACS Nano*.

[23] Yu L., Zhang Y., Zhang B., Liu J., Zhang H., Song C. (2013). Preparation and characterization of HPEI-GO/PES ultrafiltration membrane with antifouling and antibacterial properties. *Journal of Membrane Science*.

[24] Girotti S., Ferri E. N., Fumo M. G., Maiolini E. (2008). Monitoring of environmental pollutants by bioluminescent bacteria. *Analytica Chimica Acta*.

[25] Ivask A., Bondarenko O., Jepihhina N., Kahru A. (2010). Profiling of the reactive oxygen species-related ecotoxicity of CuO, ZnO, TiO_2_, silver and fullerene nanoparticles using a set of recombinant luminescent *Escherichia coli* strains: differentiating the impact of particles and solubilised metals. *Analytical and Bioanalytical Chemistry*.

[26] Deryabin D. G., Aleshina E. S., Efremova L. V. (2012). Application of the inhibition of bacterial bioluminescence test for assessment of toxicity of carbon-based nanomaterials. *Microbiology*.

[27] Jia K., Marks R. S., Ionescu R. E. (2014). Influence of carbon-based nanomaterials on lux-bioreporter *Escherichia coli*. *Talanta*.

[28] Davydov S. Y., Kryukov A. Y., Gerya V. O., Izvol'skii I. M., Rakov E. G. (2012). Preparation of a platelike carbon nanomaterial using MgO as a template. *Inorganic Materials*.

[29] Nguyen H. V., Tun N. M., Kryukov A. Y., Izvol'skii I. M., Rakov E. G. (2014). Dependence of the “solubility” of oxidized carbon nanomaterials on the acidity of aqueous solutions. *Russian Journal of Physical Chemistry A*.

[30] Kotova V. Y., Manukhov I. V., Zavilgelskii G. B. (2010). Lux-biosensors for detection of SOS-response, heat shock, and oxidative stress. *Applied Biochemistry and Microbiology*.

[31] Nikiyan H., Vasilchenko A., Deryabin D. (2010). Humidity-dependent bacterial cells functional morphometry investigations using atomic forcemicroscope. *International Journal of Microbiology*.

[32] Jastrzebska A. M., Kurtycz P., Olszyna A. R. (2012). Recent advances in graphene family materials toxicity investigations. *Journal of Nanoparticle Research*.

[33] Li D., Müller M. B., Gilje S., Kaner R. B., Wallace G. G. (2008). Processable aqueous dispersions of graphene nanosheets. *Nature Nanotechnology*.

[34] Notley S. M., Crawford R. J., Ivanova E. P., Aliofkhazraei M. (2013). Bacterial interaction with graphene particles and surfaces. *Advances in Graphene Science*.

[35] Zhang Y., Ali S. F., Dervishi E. (2010). Cytotoxicity effects of graphene and single-wall carbon nanotubes in neural phaeochromocytoma-derived pc12 cells. *ACS Nano*.

[36] Chen H., Müller M. B., Gilmore K. J., Wallace G. G., Li D. (2008). Mechanically strong, electrically conductive, and biocompatible graphene paper. *Advanced Materials*.

[37] Li J., Wang G., Zhu H. (2014). Antibacterial activity of large-area monolayer graphene film manipulated by charge transfer. *Scientific Reports*.

[38] Wu B.-S., Abdelhamid H. N., Wu H.-F. (2014). Synthesis and antibacterial activities of graphene decorated with stannous dioxide. *RSC Advances*.

[39] Wang Y.-W., Cao A., Jiang Y. (2014). Superior antibacterial activity of zinc oxide/graphene oxide composites originating from high zinc concentration localized around bacteria. *ACS Applied Materials and Interfaces*.

[40] Pretti C., Oliva M., Pietro R. D. (2014). Ecotoxicity of pristine graphene to marine organisms. *Ecotoxicology and Environmental Safety*.

